# Fano Metamaterials on Nanopedestals for Plasmon-Enhanced Infrared Spectroscopy

**DOI:** 10.1038/s41598-019-44396-9

**Published:** 2019-05-24

**Authors:** Yongseok Jung, Inyong Hwang, Jaeyeon Yu, Jihye Lee, Jun-Hyuk Choi, Jun-Ho Jeong, Joo-Yun Jung, Jongwon Lee

**Affiliations:** 1 School of Electrical and Computer Engineering, Ulsan National Institute of Science and Technology, Ulsan, 44919 Korea; 2 Nano-convergence Mechanical Systems Research Division, Korea Institute of Machine and Materials, Daejeon, 305-343 Korea

**Keywords:** Mid-infrared photonics, Optical sensors, Metamaterials

## Abstract

We report a sensing platform for surface-enhanced infrared absorption (SEIRA) spectroscopy, based on Fano metamaterials (FMMs) on dielectric nanopedestals. FMMs consist of two parallel gold (Au) nanorod antennas, with a small horizontal coupler attached to one of the nanorod antenna. When placed on SiO_2_ dielectric nanopedestals, which exhibit strong field enhancements caused by the interference between subradiant and superradiant plasmonic resonances, they provide the highly enhanced E-field intensities formed near the Au nanoantenna, which can provide more enhanced molecular detection signals. Here, the sensing characteristics of FMMs on nanopedestals structure was confirmed by comparison with FMMs on an unetched SiO_2_ substrate as a control sample. The control FMMs and the FMMs on nanopedestals were carefully designed to excite Fano resonance near the target 1-octadecanethiol (ODT) fingerprint vibrations. The FMMs were fabricated by using nanoimprint lithography and the nanopedestal structures were formed by isotropic dry-etching. The experimental reflection spectra containing the enhanced absorption signals of the ODT monolayer molecules was analyzed using temporal coupled-mode theory. The FMMs on nanopedestals achieved over 7% of reflection difference signal, which was 1.7 times higher signal than the one from the control FMMs. Based on the FMMs on nanopedestal structures proposed in this study, it may be widely applied to future spectroscopy and sensor applications requiring ultrasensitive detection capability.

## Introduction

The label-free detection of biomolecules is desirable for a multitude of applications, such as medical applications, environmental monitoring, food analysis, and general substance identifications. In the label-free detection of substances, the use of mid-infrared (mid-IR) fingerprint vibration has provided superior molecular identification capabilities by utilizing the vibrational characteristics of substances that are directly related to their molecular constituents and chemical bonds^1^. The most common method used to detect biomolecules that utilizes fingerprint vibration is Fourier transform IR (FTIR) spectroscopy; it has been applied in the various fields listed above^2^. Despite its potential, the use of this method for direct detection of thin samples, such as trace amounts of biomolecules or molecular monolayers, is limited due to the low molecular absorption characteristics in the IR region. To overcome this issue, several promising methods have recently been developed that use local near-field enhancement, associated with the excitation of plasmonic resonances in metal nanostructures such as surface-enhanced Raman scattering (SERS)^3–6^ and surface-enhanced infrared absorption (SEIRA)^7–10^. While early studies into SEIRA relied on randomly-distributed metal island films that produce broad excitation spectra^7^, recent studies based on engineered plasmonic nanoantennas or metamaterials have shown great potential for the detection and identification of minute amounts of biomolecules^10^. Large local near-field enhancement can be provided at the vibrational modes of biomolecules through properly-designed plasmonic nanoantennas, and strong mode coupling formed in between the plasmonic modes and the vibrational modes of molecules further enhances the SEIRA detection signal^10^. Various metallic nanostructures such as metallic nanorods^11–13^, split-ring-resonators^14^, fan-shaped nanoantennas^15^, metamaterial absorbers^16–19^, and nanoantennas on dielectric nanopedestals^19–21^, have recently been investigated as a new SEIRA sensing platform in which adjustable absorption and reflection properties and strong near-field enhancements can be provided. However, in general this strong local near-field enhancement has been induced in a nanometer-sized gap between metallic nanoantennas, requiring delicate fabrication processes such as electron beam lithography. Large-area patterning is therefore both difficult to fabricate and costly.

Recently, new SEIRA detection strategies based on Fano resonances have been developed for bio-sensing applications; they can obtain a sharp spectral response with high near-field intensities^22–25^. Fano resonances can be obtained using a resonator system with a specific arrangement of multiple nanoantennas, designed to support bright and dark plasmonic modes corresponding to constructive and destructive interference, respectively, at the far-field^22–26^. By employing an asymmetric arrangement of nanoantennas, a weak coupling between the two resonant modes can be introduced, allowing indirect energy transfer of incident waves to the dark resonant mode. This indirect excitation of the dark mode yields sharp transmission and reflection spectra, with high quality (Q) factors and large near-field intensities^26,27^.

Here, we introduce a sensing platform based on Fano metamaterials (FMM) on a dielectric nanopedestal, in which high near-field intensities and maximum field overlap may be provided. We use an experimental approach to determine whether FMMs on a nanopedestal may be capable of detecting analyte monolayer molecules. The structure we propose here consists of two parallel metallic nanoantennas, with a perpendicular antenna coupler attached to one of them. The two nanoantennas are positioned on top of a SiO_2_ dielectric spacer, with a smaller cross-section than the top nanoantennas, as a nanopedestal. The antenna coupler enables the indirect excitation of the dark mode, with the aim of creating Fano resonances with a sharp spectral response and a high Q-factor. The dielectric nanopedestal allows additional access to the bottom surface of the metallic nanoantenna, permitting an increased effective SEIRA sensing area as well as an integral of the total near-field intensity induced in the sensing volume determined by the sensing area and a thickness of a target molecule. In our previous work, the dielectric nanopedestals were applied to metamaterial absorbers composed of a metal-dielectric-metal layer to boost SEIRA sensing signal^19^. In this study, we first applied the dielectric nanopedestal structure to FMMs which can be designed to have a high Q-factor, one of the key elements of SEIRA detection. The Fano resonant wavelength of the FMM on the nanopedestal is mainly determined by the length of the metallic nanoantenna and by adjusting the length the Fano resonant wavelength can be tuned to fingerprint vibrational wavelengths of analyte molecules. The polarization-sensitive characteristic of the structure should also enable the accurate experimental determination of the spectral positions of the Fano resonance. We fabricate the FMM on the nanopedestal using a nanoimprint lithography process, which allows for large-area patterning and cost-efficient fabrication^28,29^. The nanopedestals supporting the metallic nanoantennas are fabricated through an isotropic dry-etching process.

## Results and Discussion

**Figure 1:**
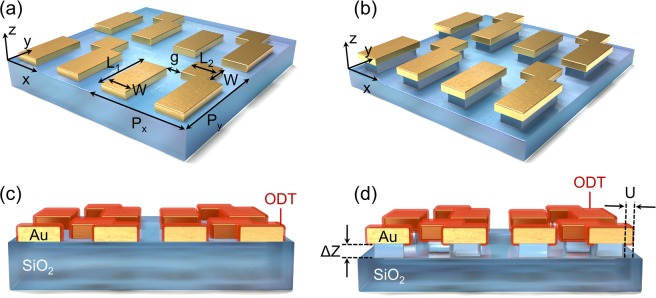
Schematics of the control FMM structure (**a**) and the FMM on the nanopedestal structure. (**b**) The dimensions of the FMM structure are: P_x_ = 1600 nm, P_y_ = 1600 nm, L_1_ = 680, 740, and 800 nm, L_2_ = L_1_/2, g = 200 nm, and W = 300 nm. Cross-sectional views of the control FMM (**c**) and the FMM on the nanopedestal (**d**) with ODT monolayer coated around the Au nanoantennas are also shown. The undercut etching depth (U) of the FMM on the nanopedestal structure is 30 nm and the vertical etching depth (ΔZ) is 50 nm.

Figure 1(a,b) illustrate the unit cell schematics of the FMMs on an un-etched SiO_2_ substrate (control) and on a SiO_2_ nanopedestal, respectively. The asymmetric configuration of the two parallel Au nanoantennas with length L_1_ along the *y*-axis, and a perpendicular Au coupler in the right nanoantenna with length L_2_ = L_1_/2 along the *x*-axis, enables the Fano resonant spectral response, in which the Fano resonant wavelength can be tuned to the target molecular vibrations. Three FMM structures with different nanoantenna lengths (L_1_ = 680, 740, and 800 nm) were designed to tune the Fano resonance excited in the structure. For the FMM on nanopedestal structure, isotropic dry etching forms a 30 nm side undercut (U) etched nanopedestal structure, which provides additional area for the analyte molecule to be coated on the bottom surface of the nanoantenna. In order to demonstrate the monolayer detection capability of the FMM on nanopedestals, we used the ODT as a target analyte molecule. It is known that the ODT molecule can form a self-assembled monolayer on an Au layer, as shown in Fig. 1(c,d)^30^. The ODT molecule has major absorption spectral peaks at 3427 nm and 3509 nm wavelengths due to the asymmetric and symmetric stretching vibration of the methylene (CH_2_) group, respectively^31,32^. The control FMM and the FMM on a nanopedestal structure were carefully designed to excite the Fano resonance near these two ODT fingerprint vibrations. For the y-polarized incident light, a dipole plasmon mode (*ω*_*D*_) was excited, and through the near field interaction between the two nanoantennas in the asymmetric configuration a quadrupole plasmon mode (*ω*_*Q*_) with a high-Q value was indirectly excited in the structure. As a result of the interference of the two plasmon modes, Fano resonance should be formed with significant near-field enhancement of the two nanoantennas. When molecular vibrations located near the Fano resonance are added to the system, the additional coupling of the molecular vibrations with the system should disturb the Fano resonance, resulting in perturbation in the far-field reflection spectrum.

**Figure 2:**
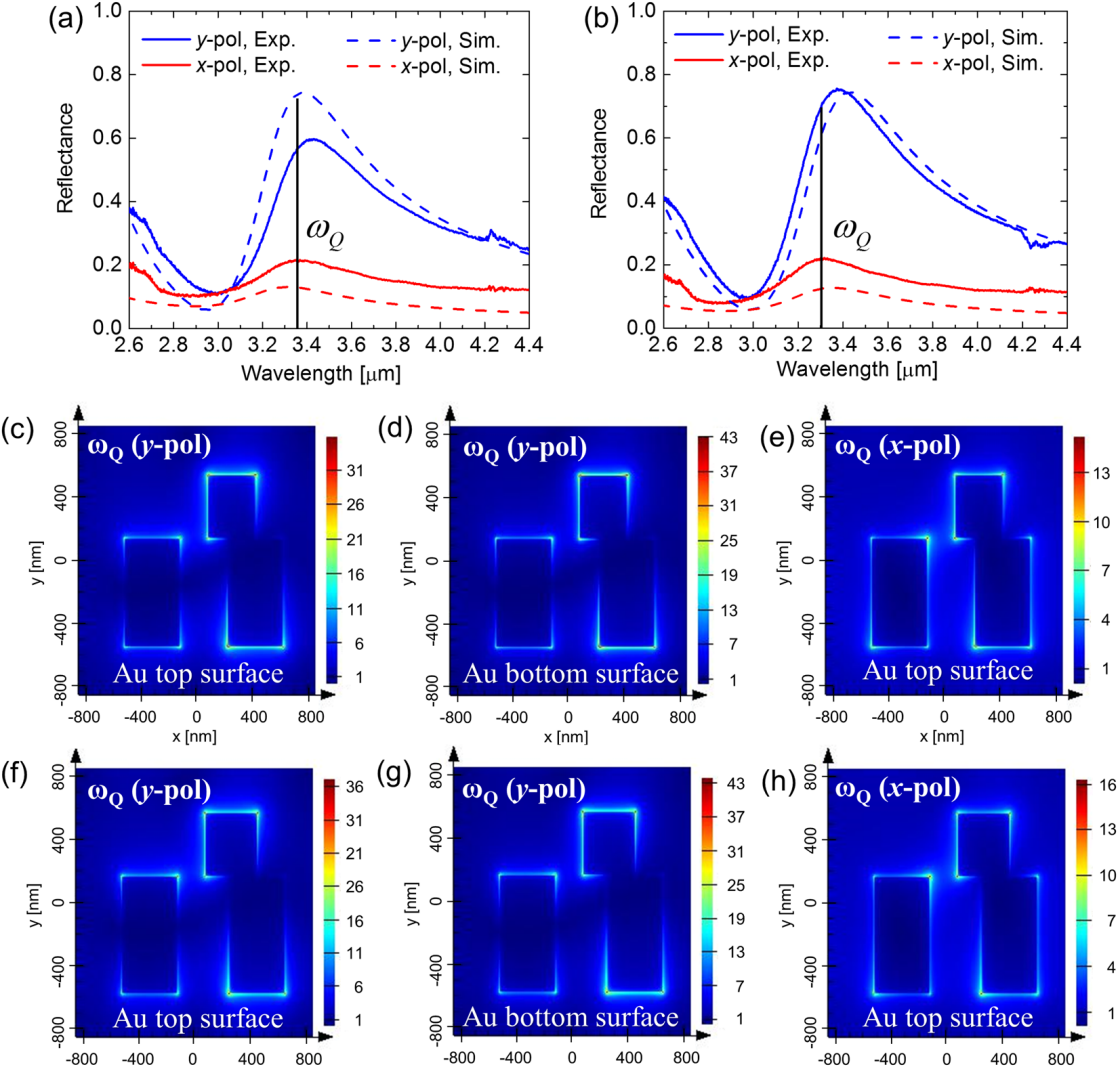
Measured (solid line) and simulated (dashed line) reflection spectrum of the bare control FMM (**a**) and the bare FMM on the nanopedestal structure (**b**) for x-polarized (red) and y-polarized (blue) incident light. The black solid line indicates the quadrupole resonance frequency of each structure. Top views of the simulated E-field enhancement distribution for the control FMM monitored (**c**) at the top and (**d**) the bottom Au surface at the quadrupole resonance frequency (λ = 3.24 μm) excited from y-polarized incident light, and (**e**) at the top Au surface at the quadrupole resonance excited from x-polarized incident light. Top views of the simulated near-field enhancement distribution for the FMM on the nanopedestal structure monitored (**f**) at the top and (**g**) at the bottom Au surface at the quadrupole resonance frequency (λ = 3.29 μm) excited from y-polarized incident light, and (**h**) at the top Au surface at the quadrupole resonance excited from x-polarized incident light.

To get a better understanding of the characteristics of the FMM on nanopedestal structure, numerical simulations were carried out (see Methods for details). To simulate the devices, the two FMM unit cells shown in Fig. 1(a,b) were used. The Fano resonant frequencies of the two structures are primarily determined by the L_1_ values of the two nanoantennas. In the asymmetric configuration of the two parallel Au nanoantennas, the Fano resonant mode is a quadrupole mode induced indirectly to the nanoantenna by *y*-polarized incident light, and the quadrupole mode having Lorentzian spectral response can be directly excited by *x*-polarized incident light. The simulated reflection spectrum of the control FMM structure with L_1_ = 680 nm and the FMM on nanopedestal with L_1_ = 740 nm for *x*- and *y*-polarized incident light are plotted in Fig. 2(a,b), respectively, exhibiting good agreement with experimental data also shown in the same figure. Both structures have asymmetric Fano reflection spectra for *y*-polarized incident light and symmetric Lorentzian reflection spectra for *x*-polarized incident light near the 3.4 μm wavelength. Figure 2(c–e) show cross-sectional profile of the field enhancement of the control FMM structure monitored at the top (Fig. 2(c,e)) and bottom (Fig. 2(d)) surface of the Au nanoantenna for *x*- (Fig. 2(e)) and *y*-polarized (Fig. 2(c,d)) incident light at quadrupole frequency, *ω*_*Q*_, indicated in Fig. 2(a). In the same way, Fig. 2(f–h) show cross-sectional profile of the near field enhancement of the FMM on the nanopedestal structure monitored at the top (Fig. 2(f,h) and bottom (Fig. 2(g)) surface of the Au nanoantenna for *x*- (Fig. 2(h)) and *y*-polarized (Fig. 2(f,g)) incident light at quadrupole frequency, *ω*_*Q*_, shown in Fig. 2(b). According to the results, the largest near field enhancement is monitored at the bottom corners of the right side nanoantenna for the Fano resonant mode indirectly induced by *y*-polarized incident light. When comparing the near-field enhancement factor induced in the two FMM structures, it turns out that a slightly higher field enhancement is induced in the FMM on the nanopedestal structure. The FMM on the nanopedestal structure provides an additional sensing area by the revealed bottom Au surface formed by undercut etching and can utilize the near field enhancement formed at the additional access, which can provide a stronger near field coupling effect with analyte molecules of interest. To quantitatively compare the two structures, the effective sensing area (revealed Au nanoantenna area) and the effective near-field intensities integrated over the sensing volume effectively covered by the 2.8 nm-thick ODT monolayer on the Au nanoantenna at 3427 nm wavelength were calculated (see Table 1). The FMM on the nanopedestal structure exhibited a 1.2 times larger sensing area and a 1.37 times higher integrated near-field intensity than the control sample, owing to the longer nanoantenna length, additionally revealed sensing surface area, and slightly higher near-field enhancement distribution of the FMM on nanopedestal structure.

**Table 1:** Calculation results of effective area for sensing and integrated E-field intensity over the sensing volume of the two FMM structures.

Case	Sensing Area [*μm*^2^]	Integrated Near-field Intensity ${\int }_{{\bf{0}}}^{{{\boldsymbol{V}}}_{{\boldsymbol{ODT}}}}{\boldsymbol{I}}/{{\boldsymbol{I}}}_{{\bf{0}}}{\boldsymbol{dV}}$ [10^9^ × nm^3^]
Control FMM structure	1.22	3.76
FMM on nanopedestal structure	1.47	5.16

30 nm of undercut etching depth was used for the FMM on the nanopedestal structure.

**Figure 3:**
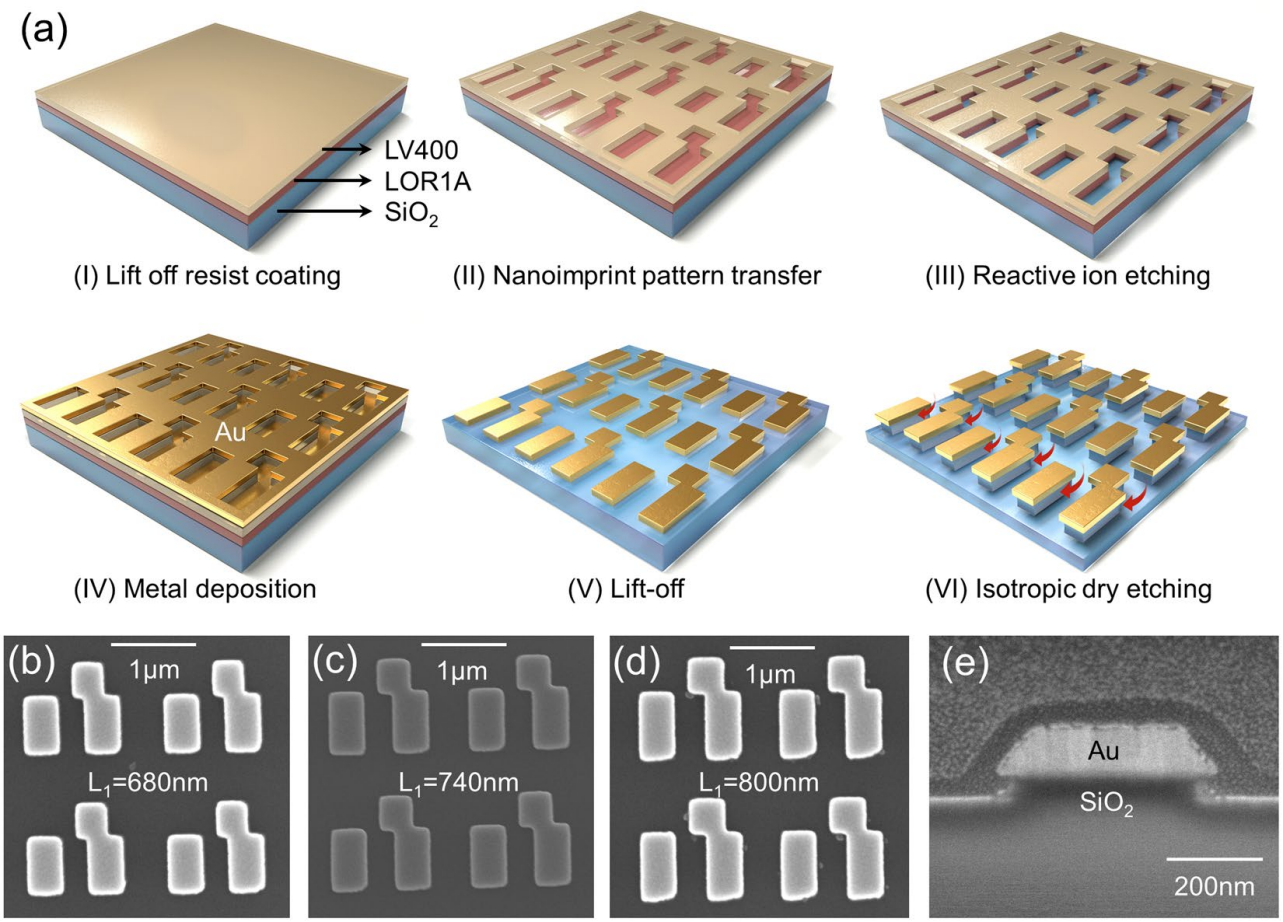
(**a**) Device fabrication process. SEM images of the fabricated FMM on the nanopedestal with (**b**) L_1_ = 680 nm, (**c**) L_1_ = 740 nm, and (**d**) L_1_ = 800 nm. (**e**) Cross-sectional side view of the FMM on the nanopedestal structure.

**Figure 4:**
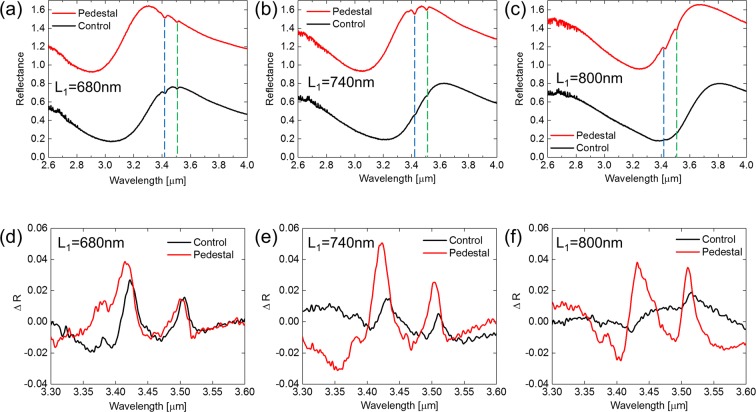
(**a**–**c**) Experimental reflection spectrum of the monolayer ODT-coated control FMM (black) and FMM on the nanopedestal (red) structures with three different nanoantenna lengths, L_1_, ((**a**) L_1_ = 680 nm, (**b**) L_1_ = 740 nm, and (**c**) L_1_ = 800 nm). The reflection spectra of the FMM on the nanopedestal structure have been given an offset of 0.8 for clarity. (**d**–**f**) Experimental reflection difference spectra of the two structures with (**d**) L_1_ = 680 nm, (**e**) L_1_ = 740 nm, and (**f**) L_1_ = 800 nm.

In order to demonstrate the biomolecule detection capability of the FMM on the nanopedestal structure, several arrays of Au FMM were fabricated on silica substrates with a 50 nm-thick layer of E-beam deposited SiO_2_ layer, using the nanoimprint lithography process. For the nanoimprint lithography, a silicon master with asymmetric parallel antennas arrays with three different sizes, as shown in Fig. 1 (caption), was made using electron-beam lithography and reactive-ion etching (RIE) process. The device fabrication step is shown in Fig. 3(a) (see Methods for the fabrication details). Three FMM arrays with different nanoantenna lengths were fabricated to study the effect of resonant frequency matching between the Fano resonant mode and ODT vibrational modes on the SEIRA detection signal. Scanning electron microscope (SEM) images of the fabricated FMM on a nanopedestal structure with three different antenna geometries are shown in Fig. 3(b–d). Figure 3(e) shows a cross-sectional view of the FMM on the nanopedestal structure prepared by focused ion-beam milling. The 30 nm undercut etching profile for the FMM on the nanopedestal structure was confirmed. For the the ODT monolayer detection experiment, six fabricated arrays (three different FMM geometries for a control FMM sample and a FMM on a nanopedestal sample) with uniformly coated ODT monolayer were prepared by immersing the samples for 24 hours in a 1m-mol ODT solution dissolved in ethanol and rinsing with ethanol to remove ODT molecules other than monolayer. Then, through the N_2_ blowing the samples were dried. Figure 4(a–c) show the measured reflection spectra of the six ODT SAM-coated FMM arrays. The black and red solid curves represent the control FMM and the FMM on the nanopedestal, respectively, and each reflection spectrum of the FMM on the nanopedestal has been offset by 0.8 for better illustration. The asymmetric Fano reflection spectra of the FMM arrays were obtained and the ODT vibrational signatures can be clearly observed for the six arrays at 3427 nm and 3509 nm wavelengths, i.e. at the ODT vibrational wavelengths. As can be seen from Fig. 4(a–c), the red-shift of the reflection spectrum becomes apparent as the length of nanoantenna increases. The Fano resonant peak and the ODT vibrational wavelengths coincided in the nanoantenna structure of L_1_ = 680 nm for the control FMM, and of L_1_ = 740 nm for the FMM on the nanopedestal structure. When the ODT vibrational wavelengths were located near the Fano resonant wavelength, the vibrational signature of the ODT in the form of the reflection deep (SEIRA signal) was measured to be the greatest, and the SEIRA signal decreased for the ODT vibrational wavelengths away from the Fano resonant wavelength. The resonance matching between the Fano resonant mode and the ODT molecular vibrational modes was crucial for inducing strong coupling between the modes, further boosting the SEIRA detection signal as a result. For better illustration of the SEIRA detection signal, the reflection difference spectrum (Δ*R* = *R*_*baseline*_ − *R*_*ODTcoated*_) is used, as shown in Fig. 4(d–f). When extracting the reflection difference spectra, we calculated baselines of the reflection spectra without the ODT vibrational signature, based on an asymmetric least square smoothing (AsLSS) algorithm. For the FMM on the nanopedestal array with L_1_ = 740 nm, 7.2% and 4% of reflection difference were observed at 3427 nm and 3509 nm wavelengths, respectively. And for the control FMM array with L_1_ = 680 nm, 4.2% and 2.5% of reflection difference were observed at 3427 nm and 3509 nm wavelengths, respectively. Comparison of the FMM on the nanopedestal structure (L_1_ = 740 nm) and the control FMM structure (L_1_ = 680 nm) both having resonance matching of the Fano resonance and ODT absorptions resulted in 1.7 times and 1.6 times improvement of reflection difference SEIRA signal at 3427 nm and 3509 nm wavelengths in the FMM on the nanopedestal structure, respectively. The improved SEIRA detection signal is attributed to the enhanced mode coupling between the Fano resonant mode and ODT vibrational modes, which originates from the larger area for sensing and the higher integrated near-field intensities of the FMM on nanopedestal structure^19^.

**Figure 5:**
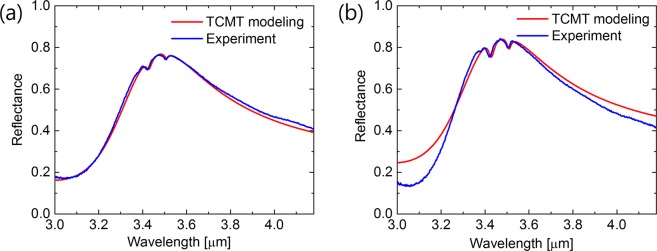
Experimental (blue) and TCMT fittings (red) of the reflection spectra for (**a**) the ODT-coated control FMM structure with L_1_ = 680 nm and (**b**) the ODT-coated FMM on the nanopedestal structure with L_1_ = 740 nm.

To examine the SEIRA signal dependence on the two FMM structures qualitatively, we used a temporal coupled-mode theory (TCMT) framework which can describe the dipole and quadrupole mode of the FMM structures as well as the two vibrational modes of the ODT molecules^22^. The dipole and quadrupole mode of the FMM and the two molecular vibrational modes of the ODT are described by the following four coupled mode equations:

1 $$\frac{dD}{dt}=j{\omega }_{D}\,D-{\gamma }_{D}\,D+j{\kappa }_{DQ}\,Q+{\alpha }_{{D}_{x}}\,{s}_{x}^{in}+{\alpha }_{{D}_{y}}\,{s}_{y}^{in},$$

2 $$\frac{dQ}{dt}=j{\omega }_{Q}\,Q-{\gamma }_{Q}\,Q+j{\kappa }_{DQ}\,D+j{\kappa }_{Q{M}_{1}}\,{M}_{1}+j{\kappa }_{Q{M}_{2}}\,{M}_{2}+{\alpha }_{{Q}_{x}}\,{s}_{x}^{in},$$

3 $$\frac{d{M}_{1}}{dt}=j{\omega }_{{M}_{1}}\,{M}_{1}-{\gamma }_{{M}_{1}}\,{M}_{1}+j{\kappa }_{Q{M}_{1}}\,Q,$$

4 $$\frac{d{M}_{2}}{dt}=j{\omega }_{{M}_{2}}\,{M}_{2}-{\gamma }_{{M}_{2}}\,{M}_{2}+j{\kappa }_{Q{M}_{1}}\,Q.$$

where *D*, *Q*, *M*_1_, *M*_2_ are mode amplitude of the dipole, quadrupole, asymmetric and symmetric CH_2_ vibrations of ODT, respectively, and they are used as subscripts in the equation to express their mode. *ω* is the resonant frequency, *γ* is the damping rate, *α* is the coupling rate between the dipole or quadrupole mode and *x*- or *y*-polarized incident wave, ${s}_{i}^{in}$ is the complex amplitude of the *i*-polarized incident wave, the *κ*_*ij*_ is the coupling rate between the mode *i* and *j*. The physical parameters (${\omega }_{{M}_{1}},\,{\omega }_{{M}_{2}},\,{\gamma }_{{M}_{1}},\,{\gamma }_{{M}_{2}}$) describing the intrinsic ODT molecule properties were taken from the ref. ^30^. Using the Eqs (1) and (2) and applying the reciprocity theorem, the reflectivity expressions for the bare FMM structure are obtained and the physical parameters (${\omega }_{D},\,{\omega }_{Q},\,{\kappa }_{DQ},\,{\alpha }_{{D}_{x}},\,{\alpha }_{{D}_{y}},\,{\alpha }_{{Q}_{x}}$) in the expressions are extracted by fitting the experimental data^22^. The reflectivity expressions for the ODT coated FMM structure are obtained by using the four equations and the details of the derivations are provided in Supplementary Data. We fitted the measured reflection spectrum of the ODT coated control FMM (L_1_ = 680 nm) and the ODT coated FMM on the nanopedestal (L_1_ = 740 nm) structure by using the reflectivity expression obtained by the TCMT modeling. Figure 5(a,b) show the reflection spectra from measurements (blue) and TCMT modeling (red) for the two FMM structures with monolayer ODT coating, respectively. The reflection spectra obtained from the TCMT modeling demonstrate a good agreement with the experimental results. We note that to match the theoretical data with the experimental data the dipole and quadrupole resonance frequencies of the ODT-coated structure have been adjusted from the bare FMM structures to compensate the frequency shifts caused by the ODT coating. All of the physical parameters that were extracted from the experimental data are provided in the Supplementary Data. Table 2 shows the results of extracted coupling rates for the two FMM structures at the two ODT vibrational wavelengths. It turns out that the 1.33 times and 1.55 times higher coupling rates are induced in the FMM on nanopedestal structure at the 3427 nm and 3509 nm of ODT vibrational wavelengths, respectively. The increased coupling rates likely result from the larger effective area for sensing and the higher integrated near-field intensity introduced in the FMM on the nanopedestal structure, as shown in Table 1. The higher coupling rate results in the larger SEIRA detection signal in the reflection spectrum of the ODT-coated FMM structure. The coupling rate induced in the ODT-coated FMM on the nanopedestal structure could be designed to have a larger value by optimizing the nanoantenna structure, and the FMM structure used in this study could also be optimized to induce a larger coupling rate, by reducing the gap between the nanoantennas.

**Table 2:** Mode coupling coefficients obtained from the theoretical fitting of the data shown in Fig. 5 for the two FMM structures at the two ODT vibrational wavelengths.

Case	*κ*_*QM*1_ [rad/s] (*λ* = 3427 nm)	*κ*_*QM*2_ [rad/s] (*λ* = 3509 nm)
Control FMM structure	2.48 × 10^12^	1.61 × 10^12^
FMM on nanopedestal	3.54 × 10^12^	2.49 × 10^12^

## Conclusion

We present a SEIRA sensing platform based on the FMM on the nanopedestal structure and experimentally demonstrate the ODT monolayer detection using the proposed structure. Over 7% of reflection difference SEIRA signal was obtained for the FMM on the nanopedestal structure, which is 1.7 times higher than the SEIRA signal obtained from the control structure. The dependence of the coupling coefficients between the quadrupole plasmon mode of the two FMM structures and vibrational modes of the ODT molecules were analyzed by a TCMT modeling. Our experimental data and the theoretical analysis of the two FMM structures together reveal the larger sensing area and the enhanced integrated near-field intensity of the FMM on the nanopedestal structure induce stronger coupling with ODT molecules, which leads a much improved SEIRA detection signal. The proposed structure and fabrication process in this work may be applied to the future development of SEIRA-based large-area sensing platform with high cost-efficiency.

## Methods

### Numerical simulation

A commercial FDTD simulation tool (Lumerical FDTD Solutions) was used to perform finite difference time domain simulations. Periodic boundary conditions in the lateral directions (x- and y-axis) and perfectly absorbing boundary condition in the vertical direction (z-axis) were placed around the unit cell structures shown in Fig. 1(a,b). The Au material parameter obtained from the Drude model with a plasma frequency of *ω*_*p*_ = 1.378 × 10^14^ rad/s and a damping frequency of Γ = 1.224 × 10^14^ rad/s was used^33^ and for the SiO_2_ material’s dielectric constant we used the built-in parameters in the software. For calculation of the integrated near-field intensities, we used a mesh refinement option with the size of 2 nm near the Au nanoantenna along all direction.

### Device fabrication

In device fabrication, a polyurethane-acrylate (PUA) mold film was prepared first, replicating the FMM pattern on the silicon master. A silica substrate with a 50 nm-thick layer of E-beam deposited SiO_2_ was also prepared. Prior to the nanoimprint lithography process, LOR1A lift-off resist (MicroChem) and LV400 resist (UV curable silicon-based resist, Chemoptics) were coated onto the SiO_2_ layer on the silica substrate. The FMM pattern arrays on the silicon master were transferred onto the bi-layer resist via the UV nanoimprint lithography process at 3 bar pressure and under UV illumination for 95 seconds. After the nanoimprint pattern transfer, a thin residual layer of the LV400 was removed by RIEusing O_2_ and CF_4_ gas mixture at 100 W of RF power and the FMM array patterned LOR1A resist layer with undercut profile was formed by O_2_ RIE at 50 W of RF power. A 3 nm-thick Cr layer (as an adhesion layer) and a 100 nm-thick Au layer were sequentially deposited on the patterned SiO_2_ layer, followed by the lift-off to form the Au FMM arrays on the SiO_2_ layer. the undercut etched SiO_2_ nanopedestal structure was formed through an isotropic dry etching using a gas mixture of 10 sccm of O_2_ and 30 sccm of CF_4_ at 300 W of RF power for 1 minute.

### Sample characterization

The reflection spectra of the FMMs coated with ODT monolayers were measured using a FTIR spectrometer (Bruker, Vertex 70) and a nitrogen-cooled MCT photodetector for *x*- and *y*-polarized incident light. The reflection spectra of the fabricated samples were collected at a resolution of 2 cm^−1^ with 64 scan averaging, and the collected spectrum was normalized to the reflection spectrum of an Au mirror.

## Supplementary information

Supplementary Information
