# Supplementary material for: Fano Metamaterials on Nanopedestals for Plasmon-Enhanced Infrared Spectroscopy
Source: Sci Rep. 2019 May 24;9:7834. doi: 10.1038/s41598-019-44396-9 (PMC6534610; doi:10.1038/s41598-019-44396-9)
Supplement: Supplementary file 1 — Supplementary Information [file 41598_2019_44396_MOESM1_ESM.doc]

**Supplementary Information for**

**Fano Metamaterials on Nanopedestals for Plasmon-Enhanced Infrared Spectroscopy**

Yongseok Jung^1^, Inyong Hwang^1^, Jaeyeon Yu^1^, Jihye Lee^2^, Jun-Hyuk Choi^2^, Jun-Ho Jeong^2^, Joo-Yun Jung^2^,*, and Jongwon Lee^1^,*

^1^ School of Electrical and Computer Engineering, Ulsan National Institute of Science and Technology, Ulsan, 44919, Korea,

^2^ Nano-convergence Mechanical Systems Research Division, Korea Institute of Machine and Materials, Daejeon, 305-343, Korea

*e-mails : [jjy2121@kimm.re.kr](mailto:jjy2121@kimm.re.kr), [jongwonlee@unist.ac.kr](mailto:jongwonlee@unist.ac.kr)

**Supplementary discussion**

**Temporal Coupled Mode Theory Model**

The reflection spectra of ODT-coated FMM structures were modeled using temporal coupled-mode theory (TCMT), which can describe how the intrinsic materials losses, external radiation losses, and coupling rates of the vibrational modes of the ODT with FMM’s plasmonic resonant mode influence the enhanced vibrational signals and their line shapes in the reflection spectra. In the TCMT, FMM structures are regarded as a single cavity with a dipole mode amplitude *D* and a quadrupole mode amplitude *Q* coupled to the input and output traveling waves (s_+_ and s-). By adding resonators of ODT vibrations with mode amplitudes *M_1_* and *M_2_* corresponding to the two ODT vibrational modes, their interactions can be described by the four coupled mode equations expressed in the main manuscript equation (1)-(4). By assuming a temporal harmonic dependence of the modes ($d/dt\to j\omega$), Equation (1)-(4) can be written in linear algebraic form, and the dipole and the quadrupole mode amplitude can be expressed as follows,

$D=\frac{\left[\left(BCD+\kappa_{QM1}^{2}D+\kappa_{QM2}^{2}C\right)\alpha_{Dx}+j\kappa_{DQ}CD\alpha_{Dx}\right]s_{x}^{in}+\left(BCD+\kappa_{QM1}^{2}D+\kappa_{QM2}^{2}C\right)\alpha_{Dy}s_{y}^{in}}{ABCD+\kappa_{QM1}^{2}D+\kappa_{QM2}^{2}C+\kappa_{DQ}^{2}CD}$ (S1)

$Q=\frac{\left(j\kappa_{DQ}CD\alpha_{Dx}+ACD\alpha_{Dx}\right)s_{x}^{in}+j\kappa_{DQ}CD\alpha_{Dy}s_{y}^{in}}{A(BCD+\kappa_{QM1}^{2}D+\kappa_{QM2}^{2}C)+\kappa_{DQ}^{2}CD}$ (S2)

where $A=j\left(\omega-\omega_{D}\right)+\gamma_{D}$, $B=j\left(\omega-\omega_{Q}\right)+\gamma_{Q}$, $C=j\left(\omega-\omega_{M_{1}}\right)+\gamma_{M_{1}}$, and $D=j\left(\omega-\omega_{M_{2}}\right)+\gamma_{M_{2}}$. Then the reflection coefficient expression of the ODT-coated FMM structure can be obtained by applying the reciprocity theorem [R1].

$r_{xx}=\frac{s_{x}^{out}}{s_{x}^{in}}=-\frac{\alpha_{Dx}D+\alpha_{Qx}Q}{s_{x}^{in}}=-\frac{\alpha_{Dx}^{2}\left(BCD+\kappa_{QM1}^{2}D+\kappa_{QM2}^{2}C\right)+\alpha_{Qx}^{2}ACD+2\alpha_{Dx}\alpha_{Qx}j\kappa_{DQ}CD}{A\left(BCD+\kappa_{QM1}^{2}D+\kappa_{QM2}^{2}C\right)+\kappa_{DQ}^{2}CD}$ (S3)

$r_{yy}=\frac{s_{y}^{out}}{s_{y}^{in}}=-\frac{\alpha_{Dy}D}{s_{y}^{in}}=-\frac{\alpha_{Dy}^{2}\left(BCD+\kappa_{QM1}^{2}D+\kappa_{QM2}^{2}C\right)}{A(BCD+\kappa_{QM1}^{2}D+\kappa_{QM2}^{2}C)+\kappa_{DQ}^{2}CD}$ (S4)

The calculated reflection spectrum from the equation (S4) is shown in the main manuscript Fig. 5 (a) and (b), and the physical parameters of TCMT modeling extracted from the measurement data are shown in Table S1 below.

**Supplementary Table 1**

|  | Control FMM with L_1_=680 nm | FMM on nanopedestal with L_1_=740 nm and U=30 nm |
| --- | --- | --- |
| $\omega_{Q} [\text{rad/s}]$ | 6.28×10^14^ | 6.30×10^14^ |
| $\omega_{D} [\text{rad/s}]$ | 7.65×10^14^ | 7.68×10^14^ |
| $\omega_{M_{1}} [\text{rad/s}]$ | 5.50×10^14^ | 5.50×10^14^ |
| $\omega_{M_{2}} [\text{rad/s}]$ | 5.37×10^14^ | 5.37×10^14^ |
| $\kappa_{DQ} [\text{rad/s}]$ | 1.36×10^14^ | 1.36×10^14^ |
| $\kappa_{QM_{1}} [\text{rad/s}]$ | 2.48×10^12^ | 3.54×10^12^ |
| $\kappa_{QM_{2}} [\text{rad/s}]$ | 1.61×10^12^ | 2.49×10^12^ |
| $\gamma_{D} [\text{rad/s}]$ | 1.38×10^14^ | 1.38×10^14^ |
| $\gamma_{Q} [\text{rad/s}]$ | 7.24×10^12^ | 7.43×10^12^ |
| $\gamma_{M_{1}} [\text{rad/s}]$ | 2.50×10^12^ | 2.50×10^12^ |
| $\gamma_{M_{2}} [\text{rad/s}]$ | 1.80×10^12^ | 1.80×10^12^ |

**Table S1**. Fitting parameters of TCMT.
